# Obesity Influences the Expression of Key Immunomodulators in Normal Human Breast Tissue, Basal-like Breast Cancer Patients, and Cell Lines

**DOI:** 10.3390/cancers14225599

**Published:** 2022-11-15

**Authors:** DeQuarius King, Muhammad G. Khodary, Temesgen Samuel, Clayton Yates, Deepa Bedi

**Affiliations:** 1 Department of Pathobiology, Tuskegee University College of Veterinary Medicine, Tuskegee, AL 36088, USA; 2 Department of Integrative Biosciences (IBS), Tuskegee University, Tuskegee, AL 36088, USA; 3 Department of Pathology, Johns Hopkins School of Medicine, Baltimore, MD 21218, USA; 4 Sidney Kimmel Comprehensive Cancer Center, Johns Hopkins University School of Medicine, Baltimore, MD 21218, USA; 5 Department of Urology, Johns Hopkins University School of Medicine, Baltimore, MD 21218, USA

**Keywords:** adipocyte-conditioned media (ACM), triple-negative breast cancer (TNBC), serum amyloid (SAA)

## Abstract

**Simple Summary:**

Exploring the connection between lifestyle and the progression and aggressiveness of some cancers has become a widely accepted approach to drug development. Metabolic and secretory functions of adipocytes in the tumor microenvironment have drawn interest to their molecular relevance in some malignancies. Adipocytes promote breast cancer growth and metastasis. Immune-sensing cascades seem to be unsuccessful in efforts to control the spread of abnormal malignant cells. Chemotherapy, radiation therapy, and immunotherapy options alone are not efficient in the maintenance of aggressive TNBC patients. There is still a major push for molecular targets to increase the efficacy of therapy options in combination with immune-response therapies. The molecular mechanism by which adipocytes promote cancer aggressiveness is not fully understood. In this study, we explored the influence of human visceral adipocyte secretory factors on immunosuppressive genes in normal breast tissue, breast cancer patients, and basal-like triple-negative breast cancer cell lines.

**Abstract:**

Among the different components of the breast cancer microenvironment are adipocytes, which are mainly composed of differentiated adipocytes and adipose progenitors. The role of obesity in tumor progression has become a key topic in clinical studies, but the mechanics of this are still misunderstood. There is significant evidence of serum amyloid (SAA1), an acute-phase protein, being heavily expressed in inflamed, septic conditions. VTCN1 and VSIR, members of the immunoglobulin family, are key players in T-cell regulation. The present study investigates the differentially expressed genes caused by adipose-conditioned media on the novel triple-negative breast cancer cell lines MDA MB 231 and MDA MB 468. RNA sequencing of adipocyte-conditioned media (ACM)-treated MDA MB 231 and MDA MB 468 cells were analyzed and compared using the gene sequencing enrichment analysis database (GSEA). GSEA was also done on microarray data from obese, non-tumorous breast tissue patients (GSE:33526) to show significantly upregulated immunomodulators. Obesity was also shown to influence gene expression related to immune sensing and evasion in a dataset analysis of basal-like obese patients (GSE:79858). We showed obesity significantly upregulated immunomodulators related to immune suppression in non-tumorous, basal-like patients, as well as in novel basal-like TNBC cell lines.

## 1. Introduction

Exploring molecular changes in cancer has grown into an insightful technique in preclinical drug development trials. Cancers that are both estrogen-negative (ER-negative), progesterone-negative (PR-negative) and Her2-negative are considered basal-like, triple-negative breast cancers (TNBC). TNBC patients are considered more prone to reoccurrence and have the worst prognosis compared to other subtypes [1]. Drug development assessments rely heavily on highly specific chemical biomarkers to increase the chances of efficacy. Recently, molecular efforts have revealed several possible therapy targets, including DNA repair markers, such as polyadenosine and ribose polymerase inhibitors (PARPi), the programmed cell death receptor (PD-L1), the epidermal growth factor (EGRF), the androgen pathway, and the NOTCH pathway [2,3], to combat the sustained progression of these aggressive subtypes. Chemotherapy and radiation therapy in combination with immunotherapy has become a promising approach for aggressive cases of cancers. In recent years, cancer mortality rates have slowly declined due to these remarkable advancements in molecular and clinical efforts. Despite these efforts, basal-like TNBC still occurs in 10–15% of patients, accounting for almost half of all breast cancer deaths, and is considered a highly aggressive and metastatic phenotype [4].

Adipokines, chemokines, and pro-inflammatory factors all play a major role in triggering innate and adaptive immune responses. Surgery, chemotherapy, radiotherapy, and target therapy are considered the major forms of anticancer treatments, but recent advances have increased the efficacy of therapy options with immunotherapy in combination with conventional therapies [5]. Enhancing antitumor adaptive immunity responses in immune-based therapy treatments has become a focus of preclinical studies. Adaptive immunity activation depends heavily on downstream innate responses that lead to pro-inflammatory IFN signaling, which should ultimately result in the elimination of pathogens. Immunity dysfunctions have been reported in many cancer patients, specifically breast cancer, melanoma, and gastrointestinal cancer, which usually results in immune evasion and, furthermore, failure of immunotherapy [6]. Pro-inflammatory responses of adaptive immunity signaling must be explored in relation to excess adipocyte secretions. Mouse- and fibroblast-derived adipose tissue cells have been explored for their adipose-like phenotypical properties in biological studies. Adipocyte-conditioned media studies give clear outlines on the characteristics of secretory factors excreted by adipocytes [7]. There has been promising evidence that adipocyte secretions trigger innate immune sensing in breast cancer [8]. It is also widely understood that macrophage polarization is specific to secretions from components in the microenvironment [9]. Cancer-associated adipocytes control several signals related to cancer progression and could influence immunosuppression and immune therapy evasion. This biological phenomenon is still yet to be fully explored. 

During inflammation, there are a host of regulatory agents that facilitate downstream biological mechanics. The acute phase protein (APP), serum amyloid A (SAA), is a highly conserved apolipoprotein associated with high-density lipoproteins (HDLs). Under inflammatory conditions, SAA accumulation facilitates the change from anti-inflammatory HDL to pro-inflammatory HDL. Studies have reported its involvement in inflammatory-related diseases, such as atherosclerosis and rheumatoid arthritis, apparently through the control of NF-κB, JNK, C/EBP, Erk/MAPK, and PI3k/Akt/mTor signaling pathways [10]. Emerging studies have also pointed towards its involvement in inflammatory-predisposed malignancies, such as breast, pancreatic, ovarian, liver, prostate, and renal carcinomas [11]. Implications in cell migration, stimulating angiogenesis, and augmenting cytokine and chemokine expression, give hope for a potential molecular driver in some cancers. Interferon (IFN) and lipopolysaccharides (LPS) have been known to activate various classical genetic pathways in macrophages [9]. Monocytic macrophages have been separated into four isoforms (M1–M4) that are broken down further based on specialized functionality. These four forms of polarization have been broken down even further to categorize their host of functions. Specifically, M2 macrophages acquire special characteristics that create immunosuppressive signaling via IL6, VISTA, IL10, and ROS expression [12]. There have also been reports on macrophages being major binding factors of SAA in the injured liver. Further differential and polarization analysis shows evidence of SAA inducing M2b-like polarization rather than M2a or M2c subtypes [11]. M2b macrophages show high IL10 and low IL12 production and are considered the more immunosuppressive isoform. 

The immune suppressive potencies of M2 macrophages are found not only in cancer, but also in other diseases associated with chronic inflammation and autoimmune disorders. It is widely understood, but not fully examined, that cancer-associated adipocytes secrete factors that are associated with tumor progression and immune signaling [13,14]. In this study, basal-like, triple-negative breast cancer (TNBC) cell lines (MDA MB 231 and MDA MB 468) were grown in human visceral adipocyte-conditioned media (ACM) and analyzed by full transcriptome RNA sequencing. We showed the influence of ACM on key immunomodulators prompting myeloid-derived suppressor cell mimicry and T-cell inhibition. ACM also significantly upregulates the expression of genes associated with further driving tumor progression. Similarly, we showed that the acute-phase protein SAA1 is significantly upregulated in obese breast-cancer patients as well as in non-cancerous breast stroma samples. In contrast, the V-set domain member VTCN1, a key T-cell inhibitor, only appears in samples from obese patients with basal-like tumors. Specifically, obesity was shown to enhance immunosuppressive genes that prime the tumor microenvironment for macrophage infiltration and inactivity. Our findings also suggest that obese patients with basal-like breast cancer are more vulnerable to immunosuppressive signatures.

## 2. Materials and Methods

### 2.1. Triple-Negative Breast Cancer Cell Lines and Culturing Procedures

All cell lines were obtained from the American Type Culture Collection (ATCC). Triple-negative phenotype breast cancer cell lines, MDA MB 231 (European-American) and MDA MB 468 (African-American), were maintained in Dulbecco’s modified Eagle’s medium (DMEM) supplemented with 10% fetal bovine serum (Sigma) and 5% penicillin-streptomycin (Sigma) at 37 °C in an incubator containing 5% CO_2_ and humidified air. 

### 2.2. Adipocyte-Conditioned Media (ACM) Collection

Preadipocytes were maintained and differentiated in 25 cm flasks and grown to an approximate 70% confluency with PBM-2 Preadipocyte growth medium-2 Bulletkit (Lonza). Differentiation was carried out based on referenced protocols [15]. Cells were grown under similar conditions as TNBC cell lines. Once lipids were formed, differentiation media was removed and 6 mL of DMEM was added to each respective flask. After 72 h, DMEM was collected from all flasks as adipocyte treatment conditioned media. 

### 2.3. Adipocyte-Conditioned Media (ACM) Sample Treatments

Cell lines were seeded at 10^5^ in 6-well culture plates and allowed to grow to 60–70% confluency. After approximately 24 h, DMEM was removed and replaced with adipocyte-conditioned media for treated samples or fresh DMEM for control samples. Cells were grown at 37 °C in an incubator containing 5% CO_2_ and humidified air. After 72 h, cell lysates were collected in 1.5 mL Eppendorf centrifuge tubes (Therno Fisher, Waltham, MA, USA). Samples were immediately packaged with dry ice and shipped to Novogene, USA (California) for RNA sequencing. Confirmation of sample viability was ensured with Novogene representatives. 

### 2.4. Bioinformatics and RNA Sequencing Data (Novogene, USA) GSEA Data Analysis

GSEA data analysis was performed on the following website: https://www.gsea-msigdb.org/gsea/doc/GSEAUserGuideFrame.html (accessed on 15 November 2021). Changes in gene expression were analyzed using Hallmark, KEGG, and REACTOME database approaches against RNA sequencing enrichment data acquired from Novogene, USA. The KEGG pathway enrichment and GO term enrichment were conducted with “Gene set enrichment analysis (GSEA)”. The GSEA considered fold-changes between groups from all the biomarkers in a pathway/GO term, whether the biomarkers were differentially expressed or not, and then calculated the enrichment score based on the magnitude of the fold changes. 

All the analyses were conducted in the R programming language V3.6.3 (R Core Team 2017). The pathway/GO over-representation and GSEA analyses were implemented with the R package clusterProfiler (Yu et al. 2012). The probe annotation was conducted with the R package AnnotationDbi and hug133a.db.

## 3. Results

### 3.1. Obesity Influences the Expression of Immunomodulators in Normal Breast Tissue Samples

The microarray of gene expression profiles of 72 normal breast tissue patients undergoing reduction mammoplasty (accession number GSE: 33526) was reviewed. Differential expression of significantly upregulated genes in obese patients compared to non-obese patients was found as shown in (Table 1). 

### 3.2. Obesity Influences the Expression of Immunomodulators in African-American and European-American Breast Cancer Patients

Next, we examined microarray data from gene expression analysis of 271 gene transcripts in 425 breast cancer patients GSE79858 in the Gene Expression Omnibus (GEO). We summarized and analyzed the distribution of BMI, race, subtype, and tumor stage as shown in (Table 2). GSEA analysis as well as logistic regression analysis were conducted, with BMI (30+ vs. <25, 25–29.99) as the dependent variable, and transcripts of reactive genes and PAM50 as independent variables. Obesity (BMI + 30%) alone did not particularly influence the expression of immunomodulators compared to non-obese patients (BMI less than 25%). Most of the cases of obese women in this dataset were of African-American (32.4%) and European (65.5%) descent (Table 2). To further confirm these findings, GSEA Reactome and KEGG data analysis tools were used to compare African-American and European-American obese breast-cancer patients (Table 3).

**Figure 1:**
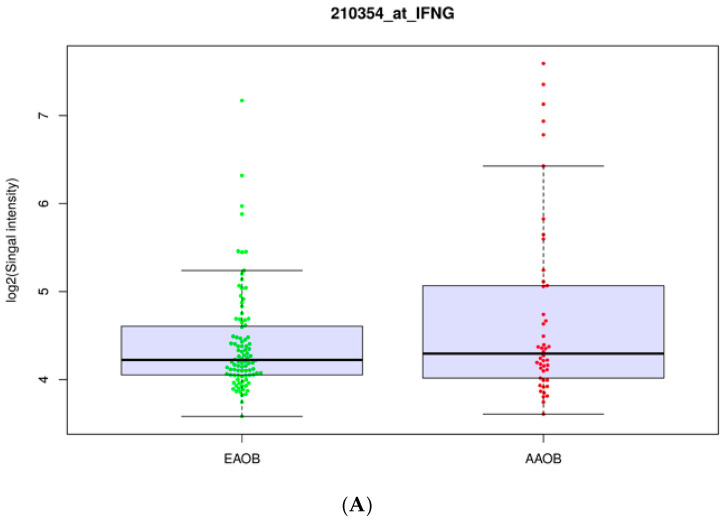

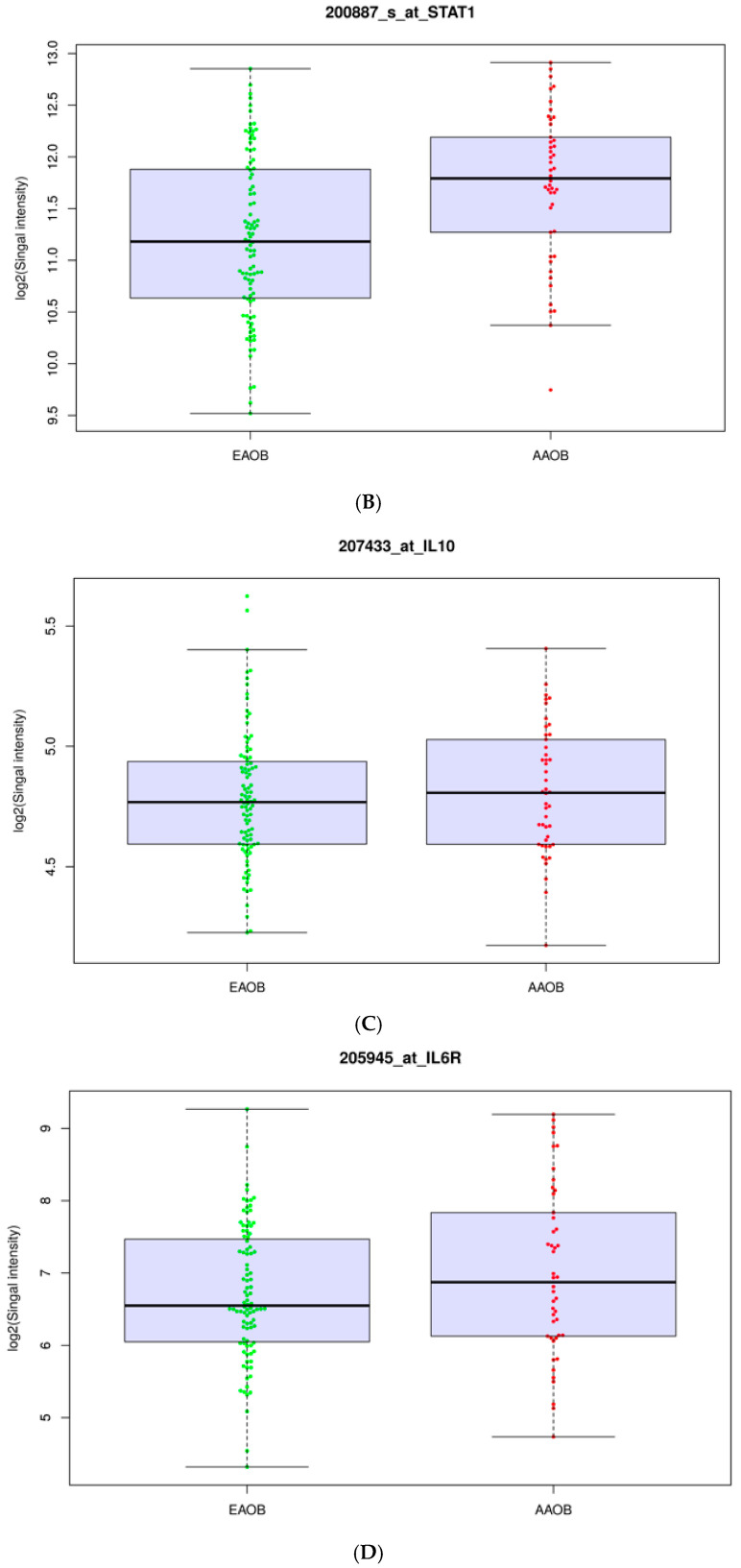
Boxplots of specific interferon signaling genes expressed in African-American obese patients compared to European-American obese patients under gene expression number GSE79858. (**A**) Boxplot of IFNG expression in obese African-Americans (AAOB) compared to obese European-Americans (EAOB). (**B**) Boxplot of STAT1 expression in AAOB vs. EAOB. (**C**) Boxplot of IL10 expression in AAOB vs. EAOB. (**D**) Boxplot of IL6R in AAOB vs. EAOB.

Differentially expressed genes in basal-like obese patients compared to obese patients suffering from Luminal B and HER2+ subtypes were confirmed. Ethnicity and subtype played a role in the influence of obesity on the gene set expressions in samples (Figure 1). African-American basal-like obese patients showed a slightly higher expression of key genes involved in interferon signaling. Obesity significantly influenced immunomodulators in basal-like obese patients (BOB) compared to other subtypes (Table 4). 

### 3.3. Human Visceral Adipocyte-Conditioned Media Significantly Upregulated Key Immunomodulators in MDA MB 231 and MDA MB 468 Cell Lines

**Figure 2:**
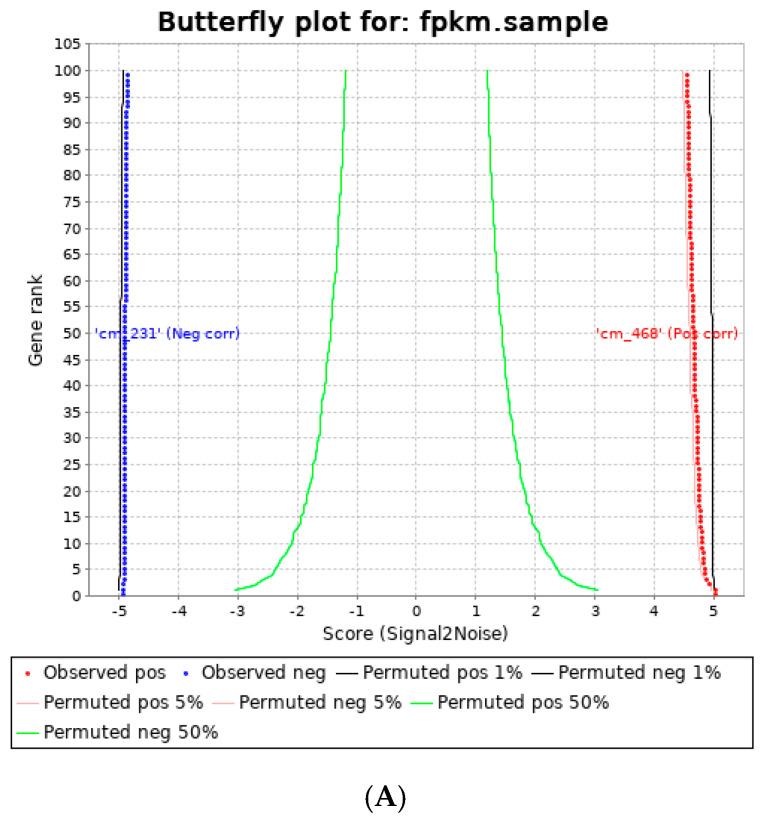

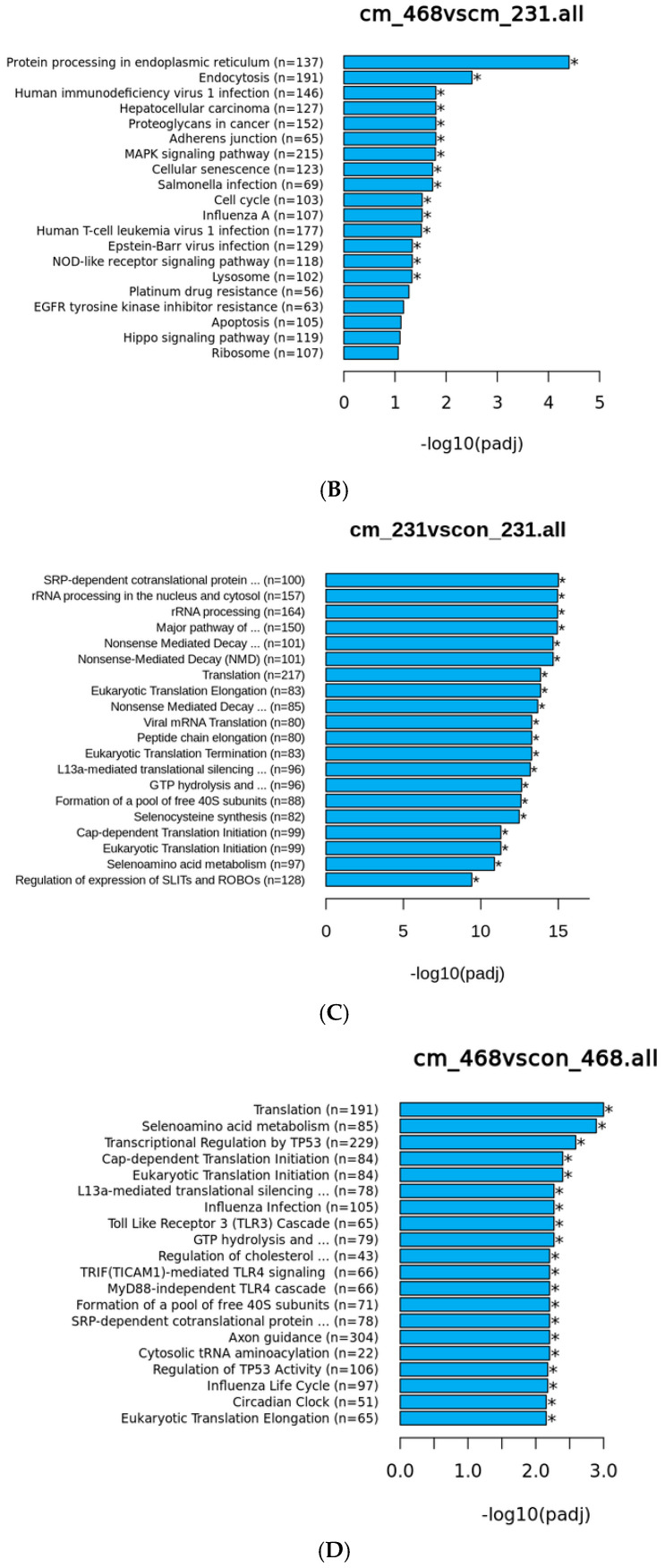
Gene ranking distribution from RNA sequencing data and REACTOME gene set enrichment data of MDA MB 231 vs. MDA MB 468 adipocyte-conditioned media samples. (**A**) Butterfly plot of gene ranking distribution for MDA MB 231 CM vs. MDA MB 468 samples. (**B**) Bar graph of gene sets significantly enriched in MDA MB 468 CM compared to MDA MB 231 CM samples. (**C**) Bar graph of gene sets significantly enriched in MDA MD 231 CM vs. control samples. (**D**) Bar graph of gene sets significantly enriched in MDA MD 468 CM vs. control samples. * significantly upregulated (*p* < 0.05).

RNA sequencing data revealed significant molecular changes in MDA MB 231 and MDA MB 468 samples treated with ACM for 72 h. Adipocyte-conditioned media (ACM) significantly influenced both MDA MB 231 and MDA MB 468 gene distribution and pathway enrichments differently. Figure 2A shows the gene ranking distribution of MDA MB 231 compared to MDA MB 468. Significantly upregulated gene sets influenced by ACM are shown in Figure 2B–D. ACM significantly upregulated several pathways in relation to the upregulation of the cell cycle, translation, toll-like receptor signaling, and other significant pathways (Figure 2). Gene set enrichment data also gave clear indications of its involvement in the upregulation of the cell cycle and transcriptional regulation. There was significant upregulation of key immune-sensing players in MDA MB 231 and this is shown in (Table 5). Adipocyte-conditioned media significantly upregulated TLR2, CXCL8, and SAA1. These are key signatures associated with poor survival in TNBC [16].

Finally, we observed the significantly upregulated genes of interest in MDA MB 468 adipocyte-conditioned media samples compared to control. There was a slightly different story for the MDA MB 468 RNA sequencing data. Molecular changes influenced by ACM were more closely related to interferon signaling. Like the GSEA of patient data, there were key interferon signaling genes being upregulated in the presence of excess adiposity in association with the African-American basal-like cell line (MDA MB 468). Genes such as JAK3, STAT3, and STAT1 were significantly upregulated in MDA MB 468 adipocyte-conditioned media samples compared to control (Table 6). Additionally, tumor suppressive genes SAA1, VSIR, and BCL3 were also significantly upregulated in ACM samples.

## 4. Discussion

Our findings give clear evidence of obese breast-tissue samples being more inclined to immunomodulatory gene expressions compared to those from non-obese patients. There was a significant upregulation of SAA1, IL10, and IL6R. SAA is an apolipoprotein of the high-density lipoprotein predominantly produced by the liver [16]. SAA is a highly conserved protein known as a uremic toxin and has been found to induce intracellular immune defense macrophage-recruiting mechanisms. It is also known that patients suffering from septic conditions exhibit elevated SAA plasma levels. The role of SAA in acute and chronic inflammation disorders has not been fully explored. Wang et al. found SAA to be a major binding factor of M2 macrophages in an injured liver. Polarized M2 macrophages (IL10+, IL12-) give rise to a host of immunosuppressive responses in the TME [17]. These macrophages, with more affinity to SAA, also produce IL1, IL6, and TNFA. It was reported that SAA1 induces the expression of CCL3, CCL2, and CXCL8 in monocytes. Gene expression analysis of non-tumorous breast stroma shows significantly high levels of IL10, IL6, and CCL2, key immunomodulators in humans (Table 1). The macrophage recruitment agents S100a8 and S100a9, were also highly expressed in obese patients. These gene signatures are considered immunosuppressive due to their involvement in monocytic trafficking and T-cell inhibition [18].

Furthermore, obese African-Americans showed a slight increase in specific gene expressions indicative of interferon signaling compared to European-Americans (Figure 1). The expression of IFNG in breast cancer patients has some inconsistent data with respect to prognosis. These data give indications that interferon signaling in obese breast cancer patients may be dependent on ethnicity. Nearly 50% of patients in this dataset with poorly differentiated cases were obese (Table 2). Luminal A was the most common subtype across all variables of BMI. Basal-like obese patients were the second most common subtype of all variables of BMI (Table 2). Interferon signaling gene sets were significantly elevated in both Hallmark and Reactome data for African-American obese patients compared to European-American obese patients. There were also significantly enriched gene sets that are related to cytokine signaling and adaptive immunity in African-American obese breast-cancer patients compared to obese European-American patients (Table 3).

Differentially expressed genes of basal-like obese patients compared to obese patients suffering from Luminal B and HER2+ subtypes were confirmed. Obesity significantly influenced immunomodulators in basal-like obese patients (BOB) compared to other subtypes (Table 4). The degree of mRNA and protein sequence homology between SAA 1 and SAA 2 make them difficult to be distinguished. The synthesis of these isoforms relies heavily on inflammatory conditioning. Re-establishing homeostasis in acute-injury phase conditions calls for the liver to produce APPs like SAA. The biological role of SAA1 and its homologous counterpart SAA2 in in vivo and in vitro leukocyte-recruiting has been linked to the activation of toll-like receptors (TLR) 2 and 4. These pattern recognition receptors are key intrinsic cytokine regulators and lead to a host of innate immune responses. TLR2 activation via SAA1 recombinantly expressed in *Escherichia coli* (*E. coli*) has been recently linked to bacterial lipoproteins. Furthermore, Immunol et al. showed that the direct activation of monocytes was dependent upon SAA1 expression without chemokine interference. However, CCL3 and CCL8 expression in monocytes rely heavily on the expression of SAA1. Obese patients with basal-like subtypes (BOB) were analyzed for their upregulated gene expressions compared to other subtypes. There was a significant upregulation of CCL8, hrSAA 1 and 2, and VTCN1. These signaling molecules are associated with immune suppression. Microarray data also showed the influence of obesity on the upregulation of STAT1, which plays a major role in interferon signaling. Obesity was also associated with significantly upregulated expression of IL6 in BOB patients. This indicates the immune signaling capabilities of obese basal-like breast cancer patients, with IL6 being a key immune response molecule.

The predisposition to inflammation for TNBC patients increases serum amyloid levels and mediates cancer progression and high mortality. Notably, high levels of VTCN1 were shown in MDA MB 231 ACM samples. This is a key regulator of T-cell activities. There was a slightly different story for the MDA MB 468 RNA sequencing data. Molecular changes influenced by ACM were more closely related to interferon signaling. Like the GSEA of patient data, there were key interferon signaling genes being upregulated in the presence of excess adiposity in association with the African-American basal-like cell line (MDA MB 468). Genes such as JAK3, STAT3, and STAT1 were significantly upregulated in MDA MB 468 adipocyte-conditioned media samples compared to control (Table 6). Additionally, the tumor suppressive genes SAA1, VSIR, and BCL3 were also significantly upregulated in ACM samples. These genes are proven to be immunosuppressive in that they promote the inactivation of key immune cells.

## 5. Conclusions

Women suffering from more aggressive breast cancers, such as basal-like triple-negative breast cancer, may show increased expressions of key immune cell modulators when this is combined with obesity.

The prognosis and aggressiveness of basal-like triple-negative breast cancer (TNBC) depends on a variety of underlying causes. Furthermore, the predisposition to low grade chronic inflammation puts obese breast cancer patients at a disadvantage when it comes to the efficacy of maintenance therapy agents. In this study, we give evidence of key suppressive immunomodulations associated with excessive adiposity. The worldwide surge in obesity has generated a push for finding disease-specific molecular targets to improve therapy options for obese TNBC patients. The development of intrinsic therapy resistance through gene expression alteration is a huge clinical problem. There are significant amounts of data positively correlating adipocyte-rich, inflamed TME and immunomodulation [7,13,19]. The clinical relevance of these data is that it delves into the underlying causes of immune-therapy evasion.

## Figures and Tables

**Table 1:** Immunomodulators highly expressed in normal breast tissue samples from obese patients.

Gene	Log2FC	*p*-Value
CCL2	0.37490968	0.0670578
*** SAA1	0.82442043	0.04869515
*** IL10	0.81295484	0.00218818
IL6	0.72002903	0.06783646
S100A8	0.55815376	0.12251586
CXCL10	0.37490968	0.0670578
S100A9	0.33124946	0.25984809
*** IL6R	0.22257634	0.04639715
FPR1	0.32516237	0.25288916
ARG1	0.19437957	0.26976972
VTCN1	−1.1666333	0.06078488

GSE33526: 72 normal breast tissue samples from patients undergoing reduction mammoplasty. Upregulated genes in obese patients (BMI > 30%) compared to mildly obese (20% > BMI < 25%) patients associated with immunomodulation. *** significantly upregulated (*p* < 0.05).

**Table 2:** Distribution of clinical characteristics in all samples.

**a. BMI by Patient Ethnicity**					
	<25	25–29.99	30+	statistic	*p*-value
African-American	22 (16.8%)	26 (19.8%)	46 (32.4%)	Fisher Exact	0.0127
Asian	6 (4.6%)	3 (2.3%)	1 (0.7%)		
European	99 (75.6%)	96 (73.3%)	93 (65.5%)		
Hispanic	3 (2.3%)	3 (2.3%)	1 (0.7%)		
Other	1 (0.8%)	3 (2.3%)	1 (0.7%)		
**b. BMI by Tumor Grade**					
	<25	25–29.99	30+	statistic	*p*-value
Moderately Diff.	4 (41.2%)	47 (35.9%)	45 (31.7%)	Fisher Exact	0.0689
Poorly Diff.	47 (35.9%)	57 (43.5%)	70 (49.3%)		
Well Diff.	29 (22.1%)	27 (20.6%)	27 (19%)		
**c. BMI by Tumor Subtypes (Via Breastprs)**					
	<25	25–29.99	30+	statistic	*p*-value
Basal-like	30 (22.9%)	34 (26%)	34 (23.9%)	Fisher Exact	0.1785
Her2-enriched	15 (11.5%)	21 (16%)	12 (8.5%)		
Luminal A	68 (51.9%)	64 (48.9%)	77 (54.2%)		
Luminal B	17 (13%)	10 (7.6%)	16 (11.3%)		
Normal-Like	1 (0.8%)	2 (1.5%)	3 (2.1%)		

**Table 3:** Gene set enrichment analysis of gene sets significantly enriched in African-American obese patients compared to European obese patients under gene expression number GSE79858.

Gene Set Name	Genes in Gene Set (K)	*p*-Value	FDR q-Value
HALLMARK_ALLOGRAFT_REJECTION	200	4.78 × 10^−11^	8.79 × 10^−8^
HALLMARK_INTERFERON_GAMMA_RESPONSE	200	8.77 × 10^−9^	8.07 × 10^−6^
REACTOME_ADAPTIVE_IMMUNE_SYSTEM	825	6.92 × 10^−8^	4.25 × 10^−5^
REACTOME_INTERFERON_GAMMA_SIGNALING	93	5.73 × 10^−7^	2.11 × 10_−4_
HALLMARK_INFLAMMATORY_RESPONSE	200	1.1 × 10^−6^	2.49 × 10^−4^
REACTOME_CYTOKINE_SIGNALING_IN_IMMUNE_SYSTEM	719	1.22 × 10^−6^	2.49 × 10^−4^
KEGG_T_CELL_RECEPTOR_SIGNALING_PATHWAY	108	1.58 × 10^−6^	2.9 × 10^−4^

**Table 4:** Immunomodulators significantly upregulated in basal-like obese breast cancer patients compared to other subtypes.

Gene	Log2FC	*p*-Value
CXCL8	1.2785982	5.12 × 10^−6^
*** VTCN1	1.07774616	1.15 × 10^−2^
*** CXADR	0.94494262	6.27 × 10^−4^
*** SAA2/SAA1	0.8243711	2.14 × 10^−3^
*** STAT1	0.79720255	6.02 × 10^−5^
*** IFNG	0.66885529	5.29 × 10^−6^
IL6R	0.56846815	3.03 × 10^−3^
CXCR4	0.29696438	9.08 × 10^−3^
IL1B	0.28383292	0.178386

GSE79858: 400 mixed population with 296 obese patients. Table shows upregulated immunomodulators in basal-like obese patients compared to non-basal-like obese subtypes. Expressions in Log2FC. *** significantly upregulated (*p* < 0.05).

**Table 5:** Immunomodulators significantly upregulated in MDA MB 231 treated with human visceral adipocyte-conditioned media.

Gene	Log2FC	*p*-Value
*** SAA1	5.65381571	N/A
*** CCL3	4.18500109	0.0072492
*** IL1A	3.42284939	3.04 × 10^−13^
*** CXCL8	3.37438802	1.29 × 10^−240^
*** VSIR	3.15589864	5.00 × 10^−285^
*** CCL5	2.71757585	1.93 × 10^−24^
*** IL6	2.68749081	2.71 × 10^−173^
VTCN1	2.629806	0.413524
CXCL2	2.53842935	1.05 × 10^−164^
*** TLR2	1.94916144	3.89 × 10^−150^
*** IL6R	1.41025426	1.02 × 10^−23^
BCL3	1.03829151	3.12 × 10^−25^
NFKB2	0.6914094	1.62 × 10^−77^
*** TLR4	0.29417262	0.01207222

RNA sequencing data of significantly upregulated immunomodulators in MDA MB 231 influenced by human visceral adipocyte-conditioned media. *** significantly upregulated (*p* < 0.05).

**Table 6:** Immunomodulators significantly upregulated in MDA MB 468 by human visceral adipocyte-conditioned media.

Gene	Log2FC	*p*-Value
*** SAA1	6.8688814	1.84 × 10^−34^
*** VSIR	4.28271469	6.55 × 10^−56^
*** SAA2	2.87594067	0.01984081
*** BCL3	1.18211525	2.44 × 10^−42^
*** JAK3	1.04059295	9.89 × 10^−14^
*** STAT3	0.88316438	1.29 × 10^−108^
IL6R	0.75315446	1.19 × 10^−13^
*** STAT1	0.73475986	9.26 × 10^−76^
*** HIF1A	0.3168464	5.28 × 10^−11^
JAK1	0.22501884	2.52 × 10^−9^
STAT2	0.16981301	0.01922399

RNA sequencing data of immunomodulators significantly upregulated in MDA MB 468 by human visceral adipocyte-conditioned media. *** significantly upregulated (*p* < 0.05).

## Data Availability

Publicly available datasets were analyzed in this study. This data can be found here: https://www.ncbi.nlm.nih.gov/geo/query/acc.cgi?acc=GSE78958, accessed on 4 October 2022.
